# Effects of probiotics on type II diabetes mellitus: a meta-analysis

**DOI:** 10.1186/s12967-020-02213-2

**Published:** 2020-01-17

**Authors:** Yun-Wen Tao, Yin-Luo Gu, Xin-Qi Mao, Lei Zhang, Yu-Fang Pei

**Affiliations:** 1 Department of Epidemiology and Health Statistics, School of Public Health, Medical College, Soochow University, 199 Ren-ai Rd., Suzhou, 215123 Jiangsu People’s Republic of China; 2 Center for Genetic Epidemiology and Genomics, School of Public Health, Medical College, Soochow University, 199 Ren-ai Rd., Suzhou, 215123 Jiangsu People’s Republic of China; 3 Jiangsu Key Laboratory of Preventive and Translational Medicine for Geriatric Diseases, Medical College, Soochow University, Suzhou, People’s Republic of China

**Keywords:** Probiotic, Gut microbiota, Type II diabetes mellitus, Meta-analysis

## Abstract

**Objective:**

The purpose of the present study was to evaluate the effectiveness of probiotics on type II diabetes mellitus (T2DM).

**Methods:**

We performed a comprehensive search on PubMed, Web of Science, China National Knowledge Infrastructure, Chinese Scientific Journal Databases, Wan Fang database and China biology medicine disc for relevant studies published before June 2019. Glycated hemoglobin A1c (HbA1c), homeostasis model assessment of insulin resistance (HOMA-IR) and fasting blood glucose (FBG) were used as indicators for T2DM. Inverse-variance weighted mean difference (WMD) with 95% confidence interval (CI) was calculated for the mean HbA1c, FBG and HOMA-IR changes from baseline.

**Results:**

15 randomized controlled trials (RCT) with a total of 902 participants were included into the meta-analysis. Considering the clinical heterogeneity caused by variation of dosage and duration of probiotic treatment, random-effects model was used to estimate the pooled WMD. Significantly greater reduction in HbA1c% (WMD = − 0.24, 95% CI [− 0.44, − 0.04], *p *= 0.02), FBG (WMD = − 0.44 mmol/L, 95% CI [− 0.74, − 0.15], *p *= 0.003) and HOMA-IR (WMD = − 1.07, 95% CI [− 1.58, − 0.56], *p *< 0.00001) were observed in probiotics treated group. Further sensitivity analysis verified the reliability and stability of our results.

**Conclusion:**

The results of our meta-analysis indicated that probiotics treatment may reduce HbA1c, FBG and insulin resistance level in T2DM patients. More clinical data and research into the mechanism of probiotics are needed to clarify the role of probiotics in T2DM.

## Background

Type II diabetes mellitus (T2DM) is a prevalent metabolic disease that have attracted wide attention because of its increasing incidence and multiple complications. T2DM is characterized by the increase of fasting blood glucose (FBG) and glycosylated hemoglobin (HbA1c), which indicate the disorder of glucose metabolism [1]. There are also a range of gastrointestinal dysfunction symptoms that occur in T2DM patients, such as delayed gastric or esophageal emptying, diabetic gastroparesis, constipation, diarrhea and obesity [2–4]. The involvement of gastrointestinal dysfunction is therefore considered as a stage in the development of T2DM [5].

The pathogenesis of T2DM is complicated and remains largely known. T2DM is considered to be a chronic inflammation [6]. Insulin resistance (IR) caused by inflammation is a characteristic feature of most patients with T2DM. T2DM is believed to be caused by a series of multiple risk factors such as genetic liability, age, overweight or obesity, and an unhealthy lifestyle. Recently, accumulated evidence suggests that the risk of developing T2DM may also involve factors from the gut microbiota. T2DM patients had a moderate degree of gut microbial dysbiosis, and the decrease of intestinal *Roseburia* and *F. prausnitzii* was detected in their stool samples [7]. As well taking the gastrointestinal symptoms of T2DM into consideration, gut microbiota is suspected to involve in the pathogenesis of T2DM.

Data from animal studies revealed that altered microbiota may contribute to the pathogenesis of IR and thereby T2DM by several mechanisms [8]. Gut microbiota mainly carry out proximal digestion of carbohydrates and ferment indigestible oligosaccharides, synthesizing short-chain fatty acids (SCFA), such as butyrate, propionate and acetate [9]. At present, the discussion on the role of SCFA is mainly considered that SCFA promotes secretion of glucagon-like peptide-1 (GLP-1) and peptide YY gastrin regulator by endocrine cells in the colon mucosa. These hormones have an influence on the gastrointestinal tract, such as inhibiting the secretion of gastric juice and gastrointestinal peristalsis, delaying the emptying of gastric contents and stimulating the hypothalamus in the central nervous system to increases the sense of satiety and appetite. Therefore, fasting blood glucose level, weight and other T2DM related indexes can be reduced [10].

Probiotics are defined as live microorganisms that, when administered in adequate amounts, confer a health benefit on the host. The data from animal studies suggested that probiotics beneficial effects can influence on glucose metabolism and improve insulin sensitivity [11]. However the effects of probiotics on human T2DM are inconsistent. Some studies showed that probiotics treatment reduced HbA1c, FBG or IR significantly in T2DM patients [12, 13], while other studies did not find significant difference between patients with probiotics and placebo [14, 15]. To evaluate the role of probiotics in T2DM patients comprehensively, and thus provide a theoretical basis for the extensive clinical application of probiotics in the treatment of T2DM, we performed a meta-analysis to evaluate the effects of probiotics on three indicators of T2DM, including HbA1c, FBG and homeostasis model assessment of IR (HOMA-IR).

## Materials and methods

### Identification of relevant studies

To identify studies with information on the effects of probiotics on T2DM, we conducted a comprehensive search of literatures published before June 2019 in PubMed, Web of Science (SCI), China National Knowledge Infrastructure (CNKI) database, Chinese Scientific Journal Databases (VIP) database, Wan Fang database and China biology medicine disc. English search terms and search patterns include: ① probiotics”, “symbiotic”, “lactobacillus”, the above search terms are linked by “OR”; ② “type 2 diabetes mellitus”, “T2DM”, or “non-insulin-dependent diabetes mellitus”; connect ① and ② with “AND”. Chinese search terms and search pattern include: ① “yishengjun”, “heshengsu”, “rusuanganjun”, the above search terms are linked with “OR”; ② “2xingtangniaobing”, “T2DM”, connect ① and ② with “AND”. Additional studies were also identified by a hand search of all the references of retrieved articles.

### Inclusion and exclusion criteria

Studies were considered eligible only if they: (1) were human clinical randomized controlled trials; (2) only included T2DM patients; (3) the intervention was probiotic, and placebo was applied as comparison to the intervention; (4) reported change from baseline to endpoint for at least one of the following outcomes: FBG, HbA1C and HOMA-IR, or could calculate by formula. Trials were excluded if: (1) subjects had recently used the probiotics or antibiotics; (2) Crucial data are incomplete; (3) studies that probably used relevant samples.

Two system evaluators browse through the literature independently and then exclude the irrelevant literature to the topic (including disease type, interventions, etc.). Read the relevant abstracts to obtain the full RCT text that might meet the inclusion criteria. The two system evaluators will check the included literature and any inconsistencies will be discussed again, or the third system evaluator will decide whether to include or not.

### Data extraction

The detailed characteristics of each trial were extracted by two system evaluators independently. The following information was extracted from each study: first author’s name, year of publication, country, dosage and duration of probiotic treatment, sample size of treatment and control groups, and effects on metabolic profiles. The data were compared and the disagreements were resolved by a third author.

### Literature quality evaluation

Trials meeting the eligibility criteria were evaluated for their quality by two researchers independently according to the Cochrane manual, which included the following six aspects: (1) generation of random distribution schemes; (2) covert grouping; (3) implementation of the blind method; (4) Integrity of the data; (5) non-selective reporting of results; (6) other sources of bias. There are three bias assessment criteria “Low”, “High”, “Unclear” for each point.

### Statistical analysis

The mean difference (MD) of change from baseline between the treatment group and control group was measured as effect size. The effect sizes and pooled estimates of the effects across the trials were calculated with RevMan 5.0 software. If the original study only provide means and standard deviations (SD) for baseline and final in each group, then the mean for change from baseline was obtained by subtracting the final mean from the baseline mean. And SD for changes from baseline was imputed using the following equation, where *R* is the correlation coefficient.

$$SD_{change} = \sqrt {SD_{baseline}^{2} + SD_{final}^{2} - 2 \times R \times SD_{baseline} \times SD_{final} }$$

The heterogeneity effects across trials was assessed using the Cochran *Q* statistic and the *I*^2^ statistic. A significance level of *p *< 0.1 or *I*^2^> 50% suggest the existence of significant heterogeneity.

The publication bias was assessed by Egger’s regression asymmetry test. A two-tailed *p* value < 0.05 was considered statistically significant. Sensitivity analysis was performed by removing one study at a time and recalculating the pooled effect size. Egger’s regression and sensitivity analysis was tested with Stata 15.0 software.

## Results

### Literature selection

**Fig. 1:**
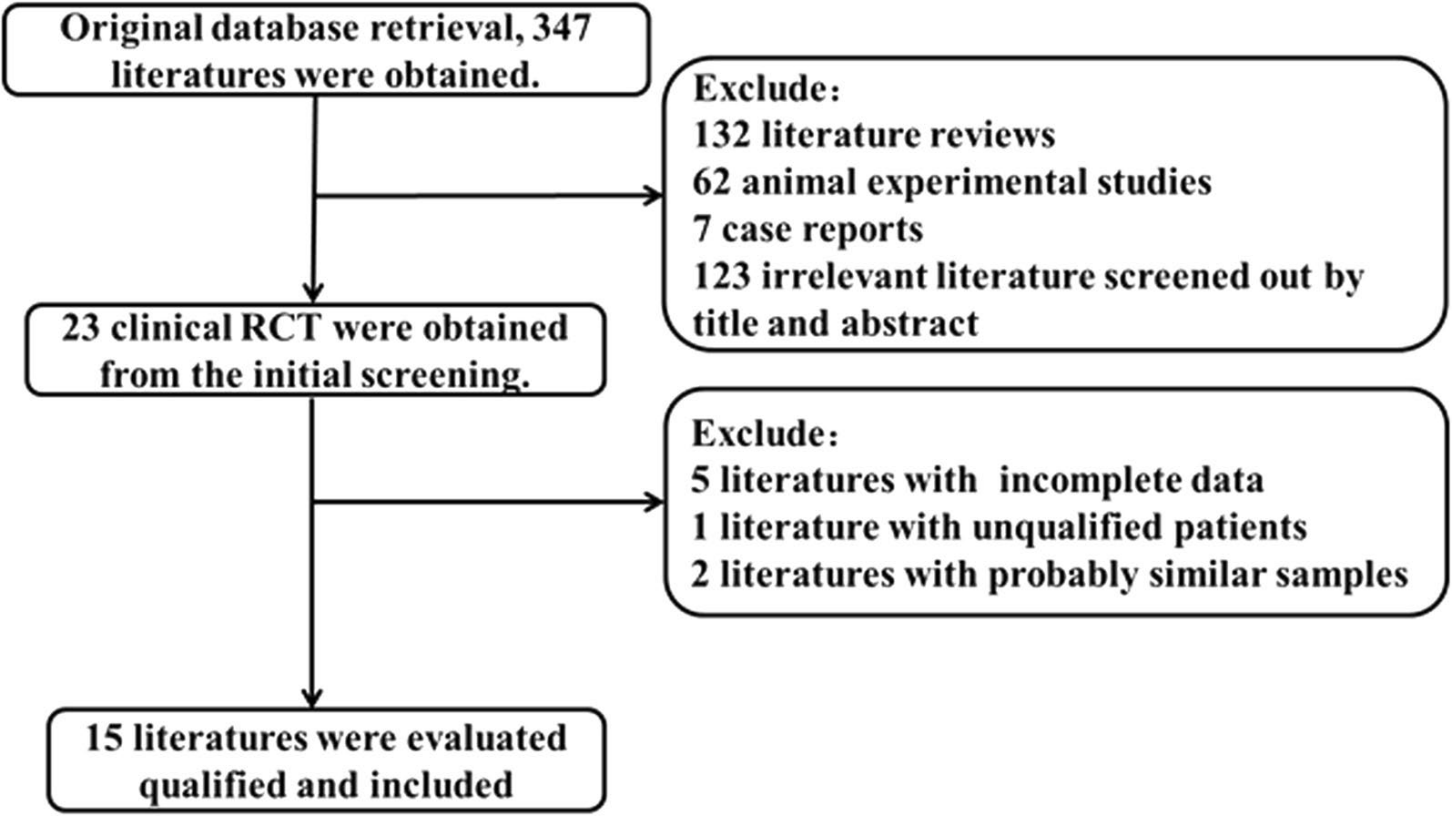
Flow chart of literature inclusion

We initially retrieved 347 relevant publications from PubMed, Web of Science, CNKI database, VIP database, Wan Fang database and China biology medicine disc. The majority of the publications were excluded because they were irrelevant studies, case reports, or reviews. According to the inclusion and exclusion criteria, 15 publications (14 in English and 1 in Chinese) with a total of 902 patients were finally included [12, 13, 15–27]. A flow chart showing the workflow for identifying and screening studies is presented in Fig. 1.

### Study characteristics

The basic characteristics of the included studies are shown in Table 1. The sample size of studies ranged from 29 to 108. The duration of treatment ranged from 6 to 12 weeks. 6 trials reported the change from baseline for HbA1c, FBG or HOMA-IR directly. While the other 9 trials only provided the mean and SD value for baseline and final, respectively. The mean and SD for change from baseline in these 9 studies were imputed with a correlation coefficient estimate of 0.5.

**Table 1:** Basic characteristics of 15 randomized controlled trials included in the meta-analysis

Study	Country	No. of cases (treatment/control)	Treatment group	Control group	Duration of treatment (week)
Razmpoosh E 2019	Iran	30/30	*Lactobacillus acidophilus* 2 × 10^9^ CFU + *Lactobacillus casei* 7 × 10^9^ CFU + *Lactobacillus rhamnosus* 1.5 × 10^9^ CFU + *Lactobacillus bulgaricus* 2 × 10^8^ CFU + *Bifidobacterium breve* 3 × 10^10^ CFU + *Bifidobacterium longum* 7 × 10^9^ CFU + *Streptococcus thermophilus* 1.5 × 10^9^ CFU 4 times/day	Placebo	6
Raygan F 2018	Iran	30/30	*Bifidobacterium bifidum* 2 × 10^9^ CFU + *La*ctobacillus *casei* 2 × 10^9^ CFU + *Lactobacillus acidophilus* 2 × 10^9^ CFU/day	Placebo	12
Ejtahed HS 2012	Iran	30/30	*Lactobacillus acidophilus La5 and Bifidobacterium lactis Bb12* 4 × 10^9^ CFU/day	Conventional yogurt	6
Shakeri H 2014	Iran	26/26	*Lactobacillus sporogenes* 1 × 10^8^ CFU/1 g 40 g 3 times/day	Bread without probiotic bacteria and prebiotic inulin	8
Khalili L 2019	Iran	20/20	*Lactobacillus casei* 1 × 10^8^ CFU/day	Placebo	8
Tajadadi-Ebrahimi M 2016	Iran	30/30	*Lactobacillus acidophilus* 2 × 10^9^ +* Lactobacillus casei* 2 × 10^9^ +* Bifidobacterium bifidum* 2 × 10^9^ CFU/g	Placebo	12
Mohamadshahi M 2014	Iran	22/22	*Lactobacillus acidophilus* 3.7 × 10^6^ CFU/g and B. lactic 3.7 × 10^6^ CFU/g 300 g/day	Conventional yogurt	8
Tajadadi-Ebrahimi M 2014	Iran	27/27	*Lactobacillus sporogenes* 1 × 10^8^ CFUs/g 3 times/day	Control bread	8
Kobyliak N 2018	Ukraine	31/22	*Lactobacillus *+ *Lactococcus* 6 × 10^10^ CFU/g + *Bifidobacterium* 1 × 10^10^ CFU/g + *Propionibacterium* 3 × 10^10^ CFU/g + *Acetobacter* 1 × 10^6^ CFU/g 10 g/day	Placebo	8
Mobini R 2016	Swedish	14/15	*Lactobacillus reuteri DSM 17938* 1 × 10^10^ CFU/day	Placebo	12
Firouzi S 2017	Malaysia	48/53	*Lactobacillus acidophilus*, *Lactobacillus casei, Lactobacillus lactis* 5 × 10^9^ CFU + *Lactobacillus acidophilus*, *Lactobacillus casei*, *Lactobacillus lactis* 5 × 10^9^ CFU 2 times/day	Placebo	12
Tonucci LB 2017	Brazil	23/22	*Lactobacillus acidophilus La*-*5* 1 × 10^9^ CFU/day + *Bifidobacterium animalis* subsp. *lactis BB*-*12* 1 × 10^9^ CFU/day	Conventional fermented milk	6
Hui Y 2018	China	53/55	*Lactobacillus paracasei N1115* 1 × 10^8^ CFU/mL 200 g 2 times/day	Placebo	8
Asemi Z 2016	Iran	51/51	*Lactobacillus sporogenes 1 *× *107* *CFU* + 0.05 g beta-carotene 3 times/day	Placebo	6
Mazloom Z 2013	Iran	16/18	*Lactobacillus acidophilus *+* Lactobacillus bulgaricus *+* Lactobacillus bifidum *+* Lactobacillus casei*	Placebo	6

### Literature quality evaluation

**Fig. 2:**
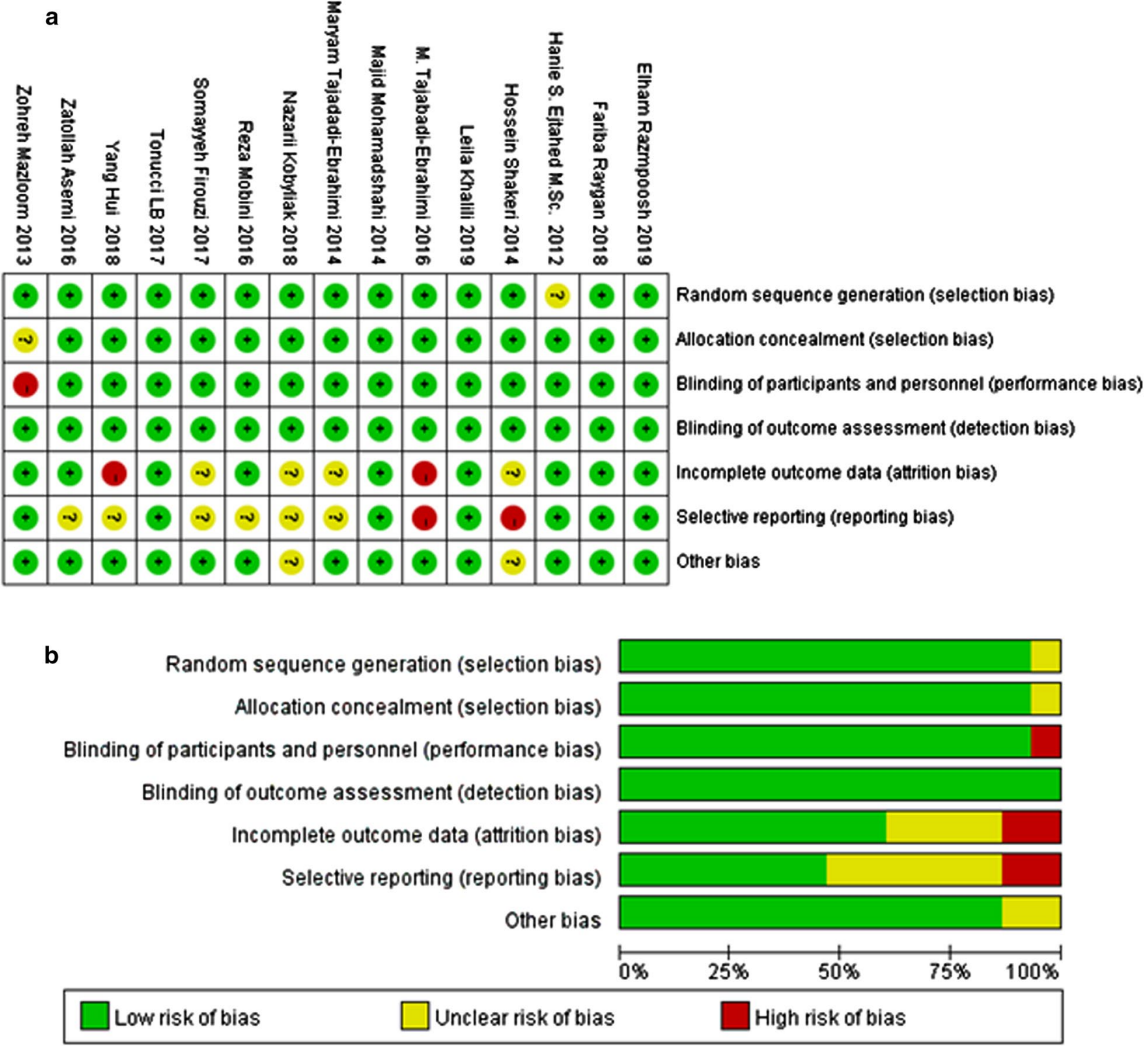
Risk of bias assessment for included studies. **a** Risk of Bias Graph. Literature quality was evaluated according to the following six points: ①generation of random distribution schemes; ②covert grouping; ③implementation of the blind method; ④Integrity of the data; ⑤non-selective reporting of results; ⑥other sources of bias. There are three bias assessment criteria “Low”, “High” and “Unclear” for each point, shown as the color green, yellow and red, respectively. **b** Risk of bias summary. The quality assessment of each literature has been shown. The color green, yellow and red represent low, high and unclear risk of bias, respectively

According to the bias risk assessment method in the Cochrane manual, all the 15 trials mentioned random sequence generation. 14 trials [12, 13, 15–23, 25–27] were double-blind clinical studies and 1 trial [24] was a single-blind study. 2 trials [20, 21] reported selecting bias. No other risk of bias was found in the 15 trials, as shown in Fig. 2. No studies were excluded according to the result of this evaluation.

### Meta-analysis

The tests for heterogeneity across the trials were performed before the trials were pooled for meta-analysis. No statistically significant heterogeneity was observed for effects of probiotics on HbA1C, FBG, and HOMA-IR (*p *= 0.22, 0.48 and 0.29, *I*^2^ = 27%, 0%, and 18%, respectively). While considering true heterogeneity caused by clinical variation, e.g. variation of treatment, dosage and duration, a random effects meta-analysis model was used to estimate the effects of probiotics on T2DM. As random-effects meta-analysis is more robust to heterogeneity effects.

### Effects of probiotics on HbA1c%

**Fig. 3:**
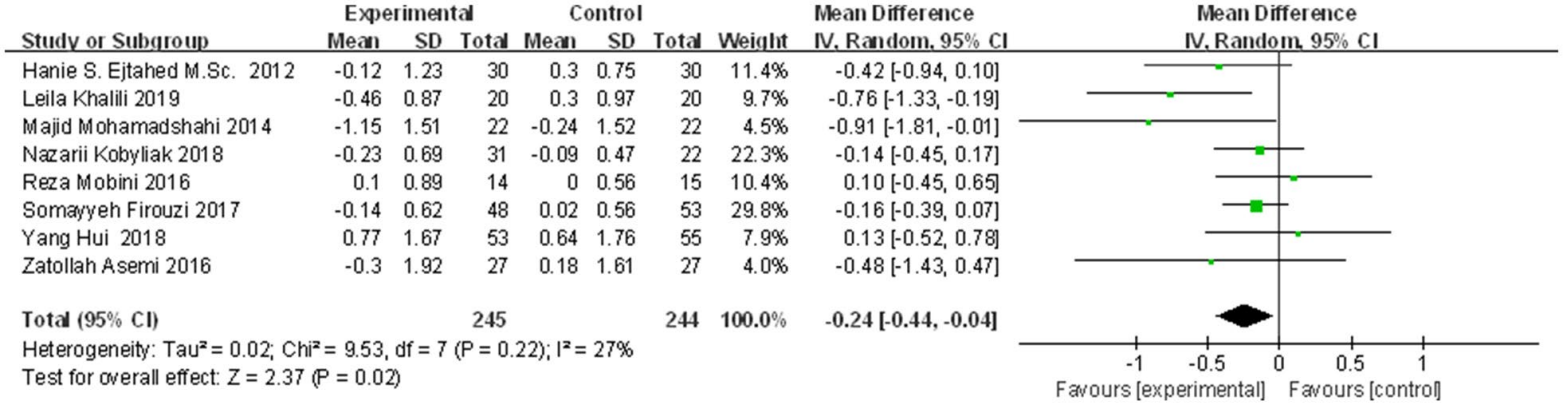
The effect of probiotics on HbA1c (%) in T2DM patients. Mean differences of change from baseline and 95% confidence intervals (CIs) are shown. Pooled estimates were calculated by the inverse-variance weighted random-effects meta-analysis. The squares indicate the effect of probiotics in a particular trial and the horizontal lines represent the corresponding 95% CIs. The diamond indicated the pooled effect size

**Fig. 4:**
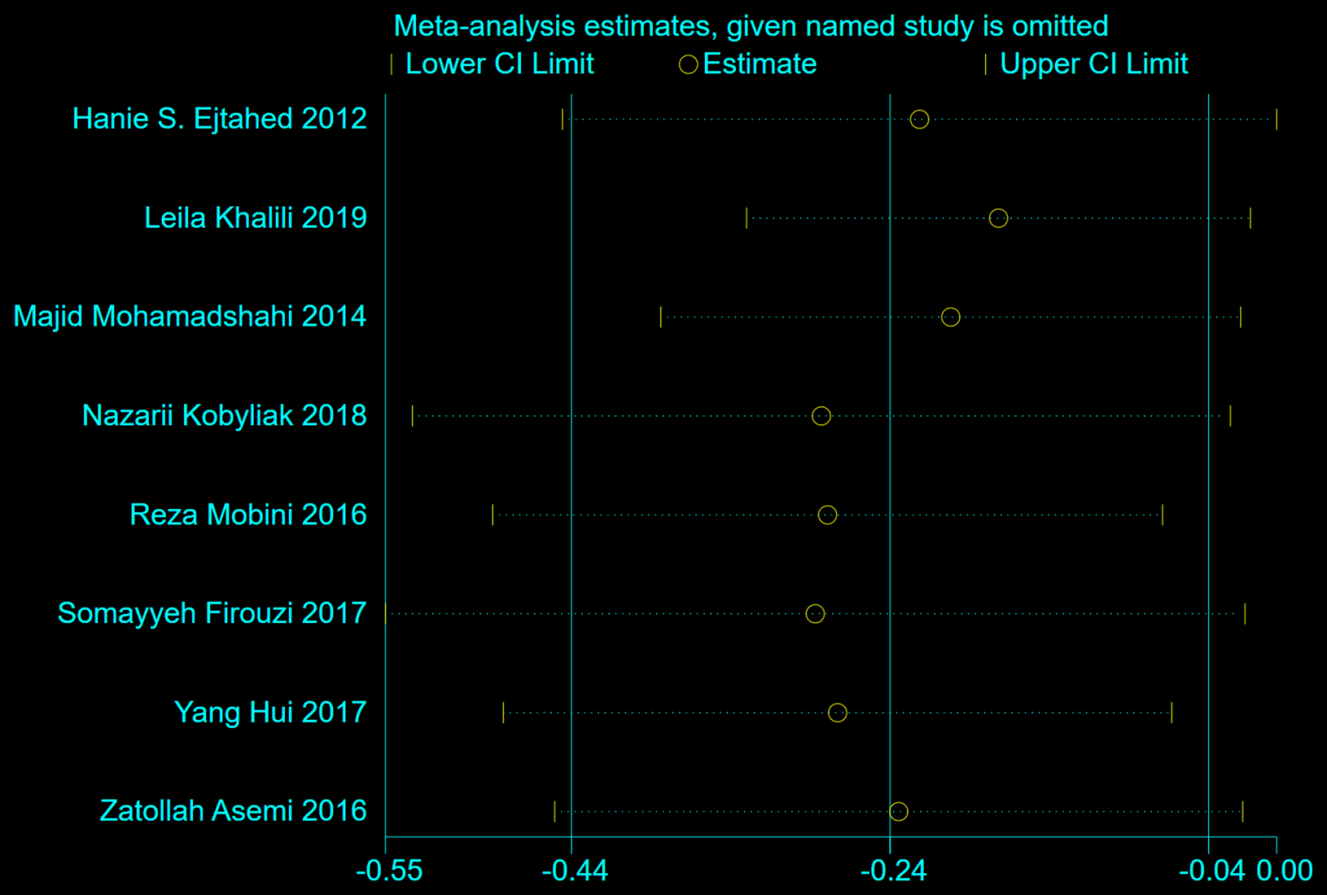
Sensitivity analysis of the effects of probiotics on HbA1c (%) in T2DM patients. Results were computed by omitting each trial in turn. Random-effects meta-analysis were used to estimate the pooled effect size. The two ends of the dotted lines represent the 95% CIs

Eight studies, with 489 participants, reported the effects of probiotics on HbA1c. The decrease in HbA1c (%) from baseline to endpoint in patients taking probiotics was statistically greater than that in control groups (inverse-variance weighted MD (WMD) = − 0.24, 95% CI [− 0.44, − 0.04], *p *= 0.02), as shown in Fig. 3. To explore the stability of this results, sensitivity analysis was performed by removing one study at a time and recalculating the pooled WMD. The results did not find substantial modification of the estimates of change in HbA1c after exclusion of any individual study, as shown in Fig. 4.

### Effects of probiotics on FBG

**Fig. 5:**
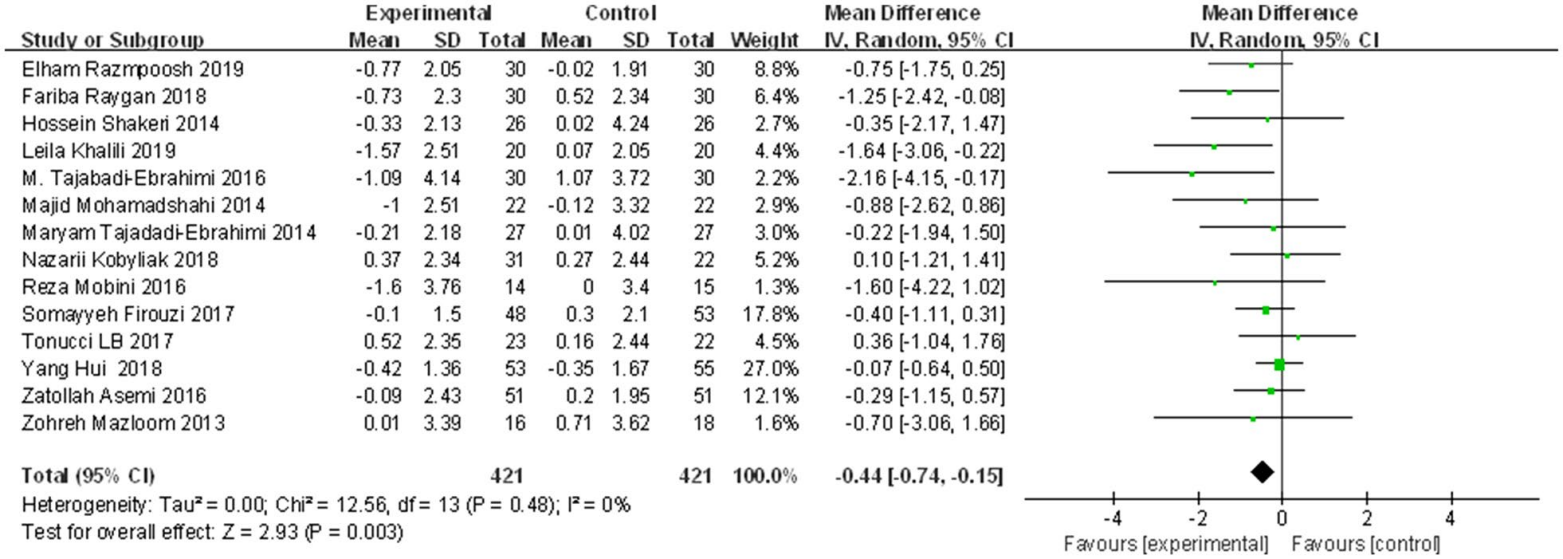
The effect of probiotics on FBG (mmol/L) in T2DM patitents. Mean differences of change from baseline and 95% confidence intervals (CIs) are shown. Pooled estimates were calculated by the inverse-variance weighted random-effects meta-analysis. The squares indicate the effect of probiotics in a particular trial and the horizontal lines represent the corresponding 95% CIs. The diamond indicated the pooled effect size

**Fig. 6:**
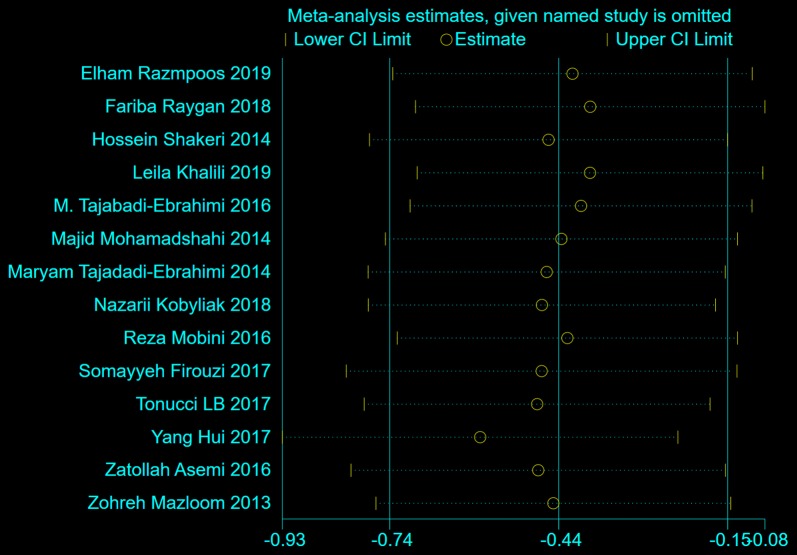
Sensitivity analysis of the effects of probiotics on FBG (mmol/L) in T2DM patients. Results were computed by omitting each trial in turn. Random-effects meta-analysis were used to estimate the pooled effect size. The two ends of the dotted lines represent the 95% CIs

The effects of probiotics on FBG was evaluated in 842 participants from fourteen trials. The results of random-effects meta-analysis in Fig. 5 showed that the difference in FBG reduction (from baseline to endpoint) between probiotics-treated groups and control groups was significant (WMD = − 0.44, 95% CI [− 0.74, − 0.15], p = 0.003). Results of sensitivity analysis demonstrated that the effects of probiotics on FBG remained consistent after removing the trials one by one, as shown in Fig. 6.

### Effects of probiotics on HOMA-IR

**Fig. 7:**
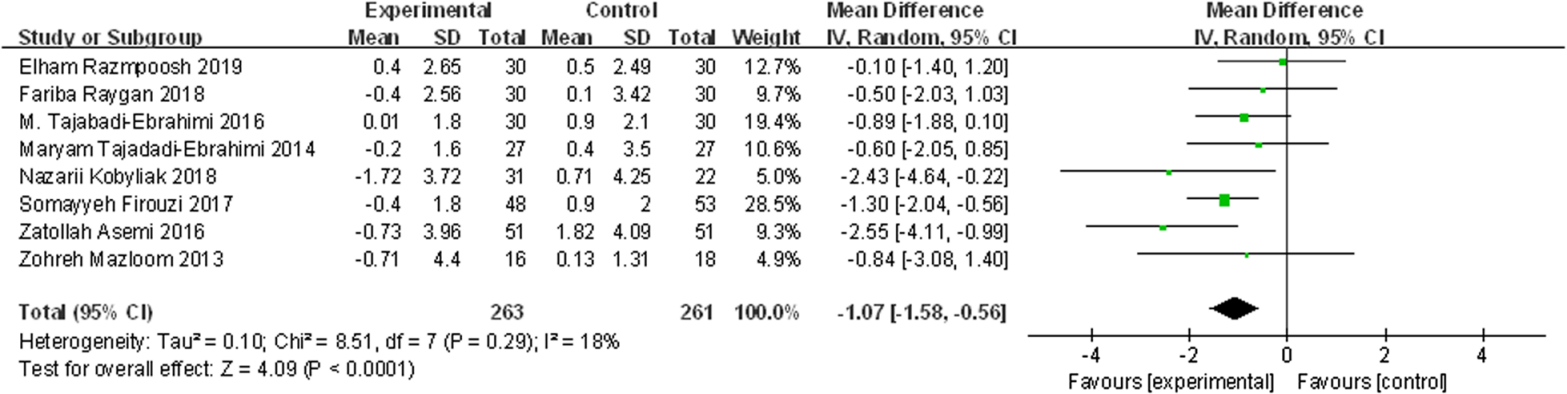
The effect of probiotics on HOMA-IR in T2DM patients. Mean differences of change from baseline and 95% confidence intervals (CIs) are shown. Pooled estimates were calculated by the inverse-variance weighted random-effects meta-analysis. The squares indicate the effect of probiotics in a particular trial and the horizontal lines represent the corresponding 95% CIs. The diamond indicated the pooled effect size

**Fig. 8:**
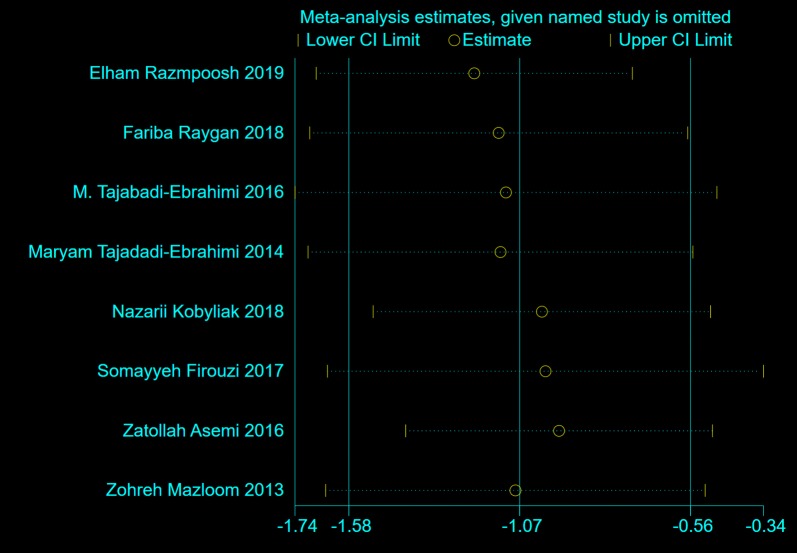
Sensitivity analysis of the effects of probiotics on HOMA-IR in T2DM patients. Results were computed by omitting each trial in turn. Random-effects meta-analysis were used to estimate the pooled effect size. The two ends of the dotted lines represent the 95% CIs

Eight trials, with 524 participants, reported the effects of probiotics on HOMA-IR. The results of the random-effects model, as shown in Fig. 7, demonstrate that the reduction in HOMA-IR (from baseline to end point) in patients taking probiotics was statistically significantly greater than that in control groups (WMD = − 1.07, 95% CI [− 1.58, − 0.56], *p *< 0.00001). Results of sensitivity analysis demonstrated that the effects of probiotics on HOMA-IR remained consistent after removing the trials one by one, as shown in Fig. 8.

### Publication bias

**Fig. 9:**
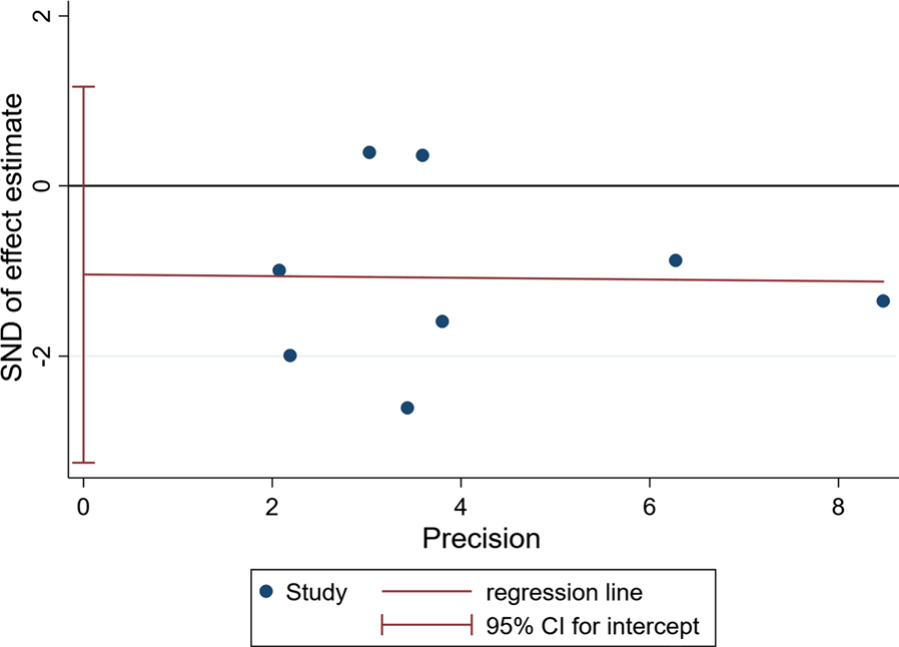
Egger’s regression asymmetry test for effects of probiotics on HbA1c% in T2DM patients. Individual studies are represented by blue dot. Success of the CI for the intercept to include zero indicates symmetry in the funnel plot and give evidence of no publication bias

**Fig. 10:**
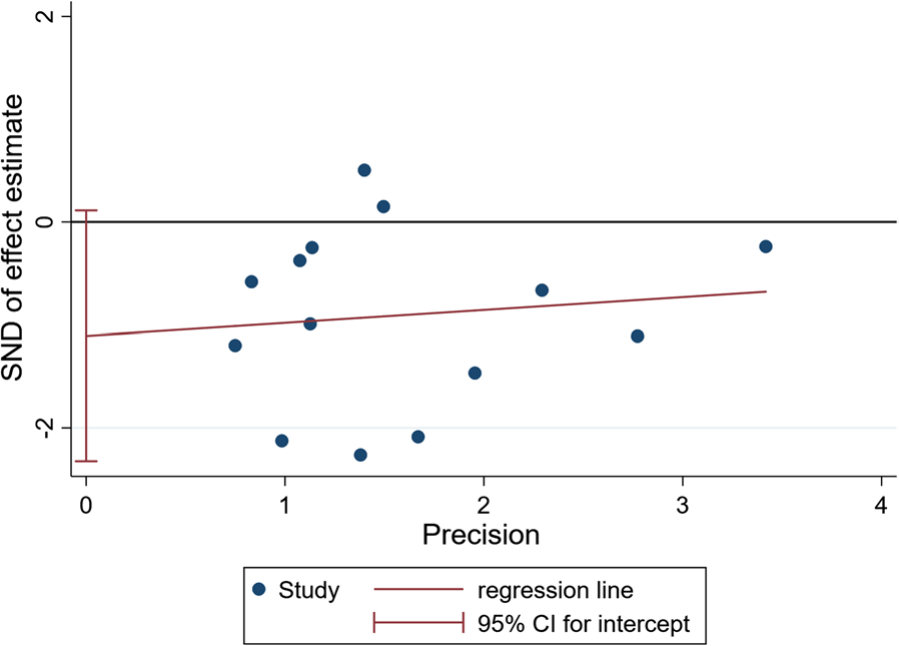
Egger’s regression asymmetry test for effects of probiotics on FBG in T2DM patients. Individual studies are represented by blue dot. Success of the CI for the intercept to include zero indicates symmetry in the funnel plot and give evidence of no publication bias

**Fig. 11:**
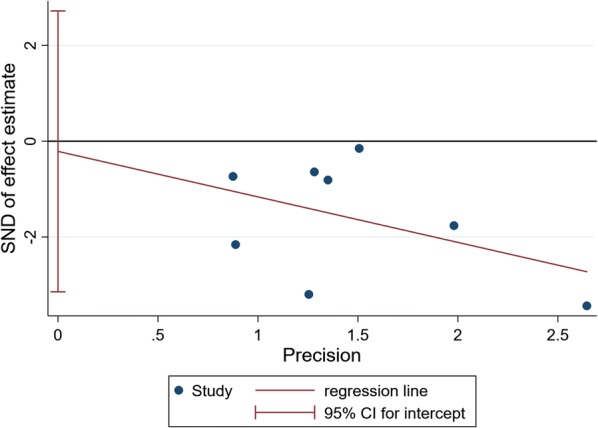
Egger’s regression asymmetry test for effects of probiotics on HOMA-IR in T2DM patients. Individual studies are represented by blue dot. Success of the CI for the intercept to include zero indicates symmetry in the funnel plot and give evidence of no publication bias

According to the publication bias tests, the effects of bias on reports of HbA1c **(**Fig. 9), FBG (Fig. 10) and HOMA-IR (Fig. 11) were not significant, as illustrated by P-values for Egger’s regression asymmetry test were 0.29, 0.86 and 0.07, respectively.

## Discussion

In the present study, we performed a systematic assessment regarding the effects of probiotics on HbA1c, FBG and HOMA-IR in T2DM patients. A total of 15 RCTs involving 902 patients were finally included into the meta-analysis. The results showed that probiotics may reduce HbA1c, FBG, and HOMA-IR levels from baseline.

The level of HbA1c in T2DM patients can precisely reflect the control of patients’ blood glucose. Both fasting and postprandial blood glucose in patients decide HbA1c level and the latter contributes significantly more to HbA1c [28]. In our meta-analysis, the effect of probiotics on HbA1c % was not as significant as that on FBG. It may be due to the unknown postprandial blood glucose level which was not included in evaluation because of insufficient clinical data. Similar findings were reported in a meta-analysis by Zhang [29].

Although probiotics improved blood sugar, accumulated evidence [12, 18] suggests that probiotics do not improve inflammation. The occurrence of insulin resistance in T2DM patients is related to obesity, however, interestingly, probiotics improved insulin resistance without changing body mass index (BMI) [12, 18]. Of note, although BMI of obese people is higher, it cannot commendably reflect the amount of body fat and inflammation in adipocytes. In Anne Sofie Andreasen’s study [30], subjects accepted *Escherichia coli* LPS (0.3 ng/kg) intravenous injection in 2 days. Before the intervention, the baseline concentrations of plasma TNF, IL-6, IL-1ra and C-reactive protein in the two groups were basically the same. However, after 4 weeks, neither probiotic treatment nor placebo treatment affected the levels of these inflammatory cytokines, suggesting that there was no direct anti-inflammatory effect of probiotics. Therefore, we speculate that the effect of probiotics on improving insulin resistance in vivo was related to the improvement of gut microbiota and the reduction of LPS translocating into blood. Moreover, the decrease of FBG and HbA1c% may result from the improvement of insulin resistance. For these reasons, we speculate that the disorder of gut microbiota in patients was not the cause of T2DM. Instead, it is more likely the product of abnormal blood glucose metabolism. Therefore, T2DM may not be cured simply relying on the treatment of probiotics.

By performing meta-analysis for RCTs, we increased the sample size and the statistical power. However, there are still some limitations. First, the number of studies included is a little small, so the validity of the results was limited. Second, our meta-analysis included several kinds of probiotics at different dosages and the therapy duration ranged from 6 to 12 weeks. Although the statistical heterogeneity across trials was not significant, true heterogeneity caused by clinical variation did exist. Third, we were unable to assess the effect of probiotics on the complications of T2DM. Thus, more RCTs with large samples are needed to confirm the effect of probiotic on glucose metabolism in T2DM patients. And experimental investigations are also urgently needed to uncover underlying mechanism of probiotics.

In summary, by meta-analyzing the effects of probiotics on HbA1c, FBG and HOMA-IR, we found that probiotics treatment can significantly improve blood glucose of T2DM patients, as reductions in all three measures were observed. Therefore, our results can provide a more comprehensive theoretical basis for the probiotic medicine using in the improvement of T2DM.

## Conclusion

In the present study, we performed a systematic assessment regarding the effects of probiotics treatment on the T2DM. A total of 15 RCT and 902 patients were included in this study. The results of our meta-analysis indicated that probiotics treatment can reduce HbA1c, FBG and insulin resistance levels in T2DM patients. The result of sensitivity analysis showed that the above results were quite robust and stable. More clinical data and research into the mechanism of probiotics are needed to clarify the role of probiotics in T2DM.

## Data Availability

The data we used can be found in the references listed.

## Abbreviations

T2DM: type II diabetes mellitus
HbA1c: glycated hemoglobin A1c
HOMA-IR: homeostasis model assessment of insulin resistance
FBG: fasting blood glucose
WMD: weighted mean difference
CI: confidence interval
SCFA: short-chain fatty acids
GLP-1: glucagon-like peptide-1
LPS: lipopolysaccharide
BMI: body mass index
