# Correction to: Effects of probiotics on type II diabetes mellitus: a meta-analysis

**DOI:** 10.1186/s12967-020-02274-3

**Published:** 2020-02-28

**Authors:** Yun-Wen Tao, Ying-Luo Gu, Xin-Qi Mao, Lei Zhang, Yu-Fang Pei

**Affiliations:** 1 Department of Epidemiology and Health Statistics, School of Public Health, Medical College, Soochow University, 199 Ren-ai Rd., Suzhou, 215123 Jiangsu People’s Republic of China; 2 Center for Genetic Epidemiology and Genomics, School of Public Health, Medical College, Soochow University, 199 Ren-ai Rd., Suzhou, 215123 Jiangsu People’s Republic of China; 3 Jiangsu Key Laboratory of Preventive and Translational Medicine for Geriatric Diseases, Medical College, Soochow University, Suzhou, People’s Republic of China

## Correction to: J Transl Med (2020) 18:30 10.1186/s12967-020-02213-2

Following publication of the original article [1], the authors reported an error in one of the author names. In this Correction the incorrect and correct author names are listed. The original publication of this article has been corrected.

Incorrect author name upon publication:

- Yin-Luo Gu

The correct author name:

- Ying-Luo Gu
